# Clinical Application of Machine Learning Models for Brain Imaging in Epilepsy: A Review

**DOI:** 10.3389/fnins.2021.684825

**Published:** 2021-06-22

**Authors:** Daichi Sone, Iman Beheshti

**Affiliations:** ^1^Department of Psychiatry, The Jikei University School of Medicine, Tokyo, Japan; ^2^Department of Clinical and Experimental Epilepsy, University College London Institute of Neurology, London, United Kingdom; ^3^Department of Human Anatomy and Cell Science, Rady Faculty of Health Sciences, Max Rady College of Medicine, University of Manitoba, Winnipeg, MB, Canada

**Keywords:** machine learning (ML), epilepsy, neuroimaging, magnetic resonance imaging, positron emission tomography (PET)

## Abstract

Epilepsy is a common neurological disorder characterized by recurrent and disabling seizures. An increasing number of clinical and experimental applications of machine learning (ML) methods for epilepsy and other neurological and psychiatric disorders are available. ML methods have the potential to provide a reliable and optimal performance for clinical diagnoses, prediction, and personalized medicine by using mathematical algorithms and computational approaches. There are now several applications of ML for epilepsy, including neuroimaging analyses. For precise and reliable clinical applications in epilepsy and neuroimaging, the diverse ML methodologies should be examined and validated. We review the clinical applications of ML models for brain imaging in epilepsy obtained from a PubMed database search in February 2021. We first present an overview of typical neuroimaging modalities and ML models used in the epilepsy studies and then focus on the existing applications of ML models for brain imaging in epilepsy based on the following clinical aspects: (i) distinguishing individuals with epilepsy from healthy controls, (ii) lateralization of the temporal lobe epilepsy focus, (iii) the identification of epileptogenic foci, (iv) the prediction of clinical outcomes, and (v) brain-age prediction. We address the practical problems and challenges described in the literature and suggest some future research directions.

## Introduction

Machine learning (ML) is an emerging trend in medicine including the fields of neurology and epileptology. The advantages of ML over conventional methods include accurate, automated, and fast pattern learning, which can be used to develop and/or optimize clinically useful algorithms for clinical medicine and basic research.

Epilepsy is a common neurological disease characterized by recurrent seizures associated with abnormal neuronal activities in the brain. Approximately 50 million people suffer from epilepsy worldwide, with symptoms that range from recurrent seizures and their physical problems to various psychosocial and psychiatric comorbidities (Collaborators, 2019). To better treat patients with epilepsy, appropriate seizure management and therapies for other aspects of epilepsy are important. However, there is a certain level of heterogeneity in epilepsy, which may prevent the best treatment for each individual patient (Pitkanen et al., 2016). ML methods could potentially outperform conventional approaches in terms of optimizing clinical diagnoses, prediction, and personalized medicine.

Recent clinical and experimental applications of ML for epilepsy include automatic seizure detection from clinical data, pre-surgical planning, the prediction of medical and surgical outcomes, and automated neuroimaging analyses (Abbasi and Goldenholz, 2019). Neuroimaging is one of the clinically essential exams for epilepsy (Bernasconi et al., 2019). While the main role of neuroimaging in epilepsy is the detection of the focus lesion in drug-resistant epilepsy, there is promising evidence of further usefulness of neuroimaging, such as the prediction of cognitive functions and postsurgical seizure outcomes in epilepsy (Bernasconi and Wang, 2021). In addition, ML methods usually require “big data” from multiple databases to provide reliable results, and in fact the development of ML has been driven by improved data collection, storage, and processing (Abbasi and Goldenholz, 2019). In this regard, neuroimaging may have some advantages for data sharing, since it has standardized protocols across various institutes and covers essentially the whole brain. The neuroimaging modalities MRI and PET are widely used in clinical practice and have been thoroughly investigated. Given the rapid development in neuroimaging techniques and ML, both of these can be expected to continue to further progress interactively. Thus, to efficiently understand and promote such development, it is meaningful to thoroughly review the current literature on ML applications for neuroimaging in epilepsy.

In this review, we have focused mainly on the existing applications of ML for brain MRI (including structural, diffusion, and functional MRI) and PET in epilepsy, aiming to provide an at-a-glance overview of these modalities. We first present a brief overview of neuroimaging modalities and ML models that are commonly used in epilepsy, such as data reduction/feature selection, classification/regression, and validation methods. We then provide a comprehensive review of the state-of-the-art ML models for epilepsy in clinical settings. To this end, we considered the following clinical aspects related to applications of ML models for brain imaging in the field of epilepsy: (i) the differentiation of individuals with epilepsy from healthy controls, (ii) the lateralization of the temporal lobe epilepsy focus, (iii) identifying the epileptogenic foci, (iv) the prediction of clinical outcomes, and (v) brain-age estimation. Lastly, we address the challenges and limitations of the existing studies, and we present potential future lines of research in this field.

## Literature Search and Study Selection

**FIGURE 1:**
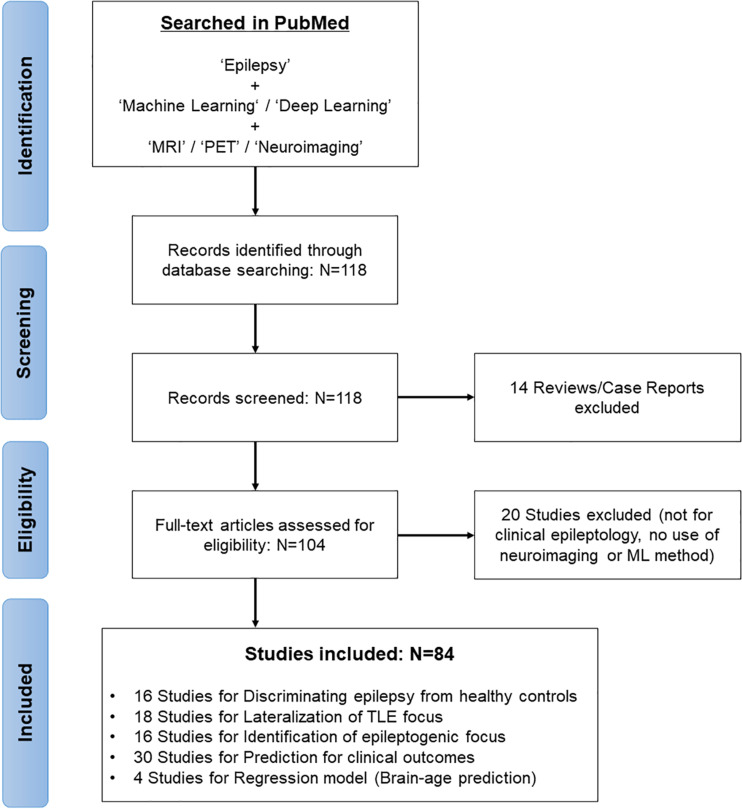
The search and inclusion of research papers in this review along with a PRISMA diagram.

In February 2021, we systematically reviewed the relevant articles in the PubMed database by first performing a literature search concentrated on the application of ML models for brain imaging in epilepsy along with a Preferred Reporting Items for Systematic Reviews and Meta-Analyses (PRISMA) diagram (Moher et al., 2009; Figure 1). The search strategy used “Epilepsy” combined with the following terms: “machine learning,” “deep learning,” “MRI,” “PET,” and “neuroimaging.” The search yielded 118 studies, of which we excluded 14 reviews and case reports at the initial screening. Studies not focusing on clinical epileptology, neuroimaging, or machine learning were also excluded from the review (*n* = 20). A final total of 84 studies were reviewed, based on the study purposes, participants, imaging modalities, feature extractions, and ML models in epilepsy (Figure 1).

## From Neuroimaging to Prediction Frameworks: An Overview

### Neuroimaging Modalities in Epilepsy

The typical structural brain MRI modalities in epilepsy include T1-weighted images (T1WI), T2-weighted images (T2WI), and fluid-attenuated inversion recovery (FLAIR), which are recommended as standard clinical protocols for epilepsy (Bernasconi et al., 2019). T1WI is used for evaluations of brain morphology. The cortical thickness of each gyrus and the volumes of each brain structure, e.g., hippocampus, can be calculated using T1WI, which has been frequently used for ML analyses. T2WI is useful to evaluate hippocampal internal structures, the amygdala, and parahippocampal cortices, while the FLAIR image sequence is suitable for the detection of focal cortical dysplasia type II, which frequently shows hyperintense FLAIR signals (Bernasconi et al., 2019). Diffusion MRI is also widely investigated in epilepsy, particularly when the white matter tract integrity in the brain is examined (Otte et al., 2012). Diffusion tensor imaging (DTI) metrics, such as fractional anisotropy (FA) and mean diffusivity (MD), have been conventionally utilized for white matter evaluations as well as ML applications. Multi-shell protocols of diffusion MRI including diffusion kurtosis imaging (DKI) and neurite orientation dispersion and density imaging (NODDI) have provided further information on brain microstructures (Jensen et al., 2005; Zhang et al., 2012). In addition to microstructural evaluations, brain structural networks can be measured by diffusion MRI.

**FIGURE 2:**
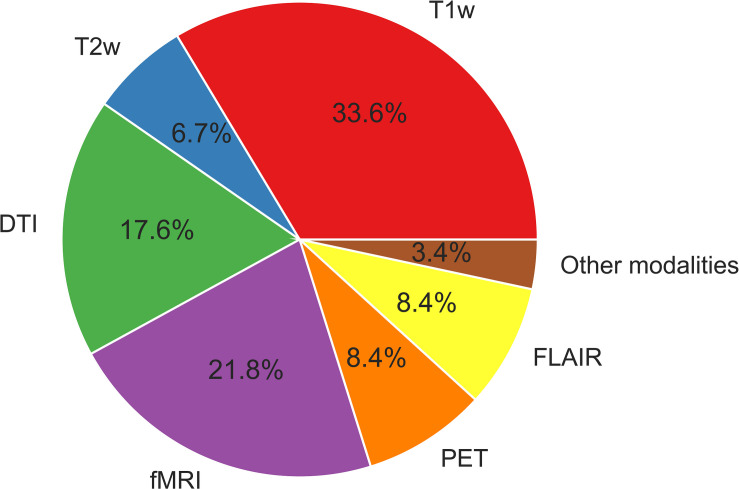
The neuroimaging modalities used in machine learning-based epilepsy studies.

Functional MRI provides information on hemodynamic brain activities by measuring blood oxygen level-dependent (BOLD) signals. Resting-state BOLD signals have recently been used to evaluate brain functional networks; in addition, metrics derived from resting-state functional MRI (e.g., functional connectivity, regional homogeneity, and the amplitude of low-frequency fluctuation) are sometimes used for ML analyses. 18F-fluorodeoxyglucose (FDG)-PET is an established examination for epilepsy, as it shows reduced signals around epileptogenic foci reflecting abnormal glucose metabolisms (Kumar and Chugani, 2013). FDG-PET signals thus indicate brain regional metabolisms and are sometimes used for a ML analysis in epilepsy. The uses of the different neuroimaging modalities described in this review are depicted in Figure 2. As can be seen in Figure 2, T1WI measurements have been used the most widely in machine learning-based epilepsy studies, probably due to the availability of plentiful T1WI brain scan data.

For the uses of machine learning, we need to extract features from the imaging modalities, and as described above, morphological features from T1WI, signal intensity from T2WI or FLAIR, diffusion metrics (e.g., FA, MD) from DTI, connectivity metrics from functional MRI, or glycometabolism data from FDG-PET are commonly used for feature extraction in machine learning. The feature extraction technique and the imaging modality are crucial factors for successful ML classification as well as easier and wider clinical applications.

### Feature Selection and Data Reduction

The raw feature space in neuroimaging data is generally much greater than the number of samples, particularly for voxel-based feature extraction strategies. The main objectives of feature-selection/data-reduction methods are avoiding both the “curse of dimensionality” and overfitting, and selecting the most informative feature sets. The aim of feature reduction algorithms is to represent a lower dimensional space of the high-dimensional original data. Among the different data reduction methods, the principal component analysis (PCA) has been widely used in epilepsy studies (Beheshti et al., 2020c; Sone et al., 2021). It should be noted that the PCA method is categorized as an unsupervised technique which only reduces the input space without improving the prediction accuracy.

The main objectives of feature-selection methods are (i) exploring the features that are relevant to the specific ML task, (ii) selecting the most informative features, and (iii) improving the prediction accuracy. Various feature-selection methods have been used in the field of epilepsy, including feature ranking (Vasta et al., 2018; Beheshti et al., 2020b), analysis of variance (ANOVA)-based feature selection (Cantor-Rivera et al., 2015), correlation-based feature selection (Cantor-Rivera et al., 2015), the F-score, least absolute shrinkage and a selection operator, and mutual information (Vasta et al., 2018).

### ML Models

The aim of a ML model is to explore a pattern between a dependent variable and corresponding independent variables in the training dataset (after feature reduction/selection) to determine a predicted status (or value) on test datasets (i.e., unseen data). The following is a brief discussion of ML algorithms (i.e., classification and regression models) used in existing epilepsy studies.

#### Classification Models

The most frequently used classification techniques in epilepsy are a support vector machine (SVM), neural networks (NN), random forest, and deep learning. An SVM is the powerful classifier based on statistical learning principles, and the SVM technique has been widely used for epilepsy classification tasks. During the training phase, an SVM finds the best class separating a hyperplane, which contributes to the maximum margin between classes. An SVM with a linear kernel can be used for linearly separable data, whereas non-linear kernel transformations such as quadratic, polynomial, and radial basis function (RBF) kernels can be used for non-linearly separable data. For example, Beheshti et al. (2020a) used an SVM classifier with a linear kernel for the classification and lateralization of MRI-negative temporal lobe epilepsy (TLE) based on FLAIR data.

An artificial neural network (ANN) is a compactional model based on biological neural networks that compose the human brain. An ANN is formed based on a set of layers (i.e., layer, one or more hidden layers, and an output layer) that are independent of each other, plus connected nodes that are called “artificial neurons.” The number of nodes in each layer is arbitrary. In an AAN structure, each node is connected to every other node and each connection has a weight and threshold. Different ANN structures have been used for brain imaging data in epilepsy (Kerr et al., 2013a, b; Pedersen et al., 2015). For example, Kerr et al. (2013b) used a multilayer perceptron (MLP) model as a classifier for the diagnosis and localization of lateralized TLE. In that study, the authors compared the SVM algorithm with a feed-forward multi-layer persectron neural network (MLPNN) for the lateralization of epileptogenic hippocampus based on MRI data.

Deep learning is a set of machine-leaning algorithms (essentially a neural network with three or more layers) that is able to learn features from the data in order to reach a high degree of abstraction (Plis et al., 2014). Deep learning embeds the feature-extraction stage in the learning phase (Shen et al., 2017). Although deep-learning methods have attracted much attention in neuroimaging studies (Zhang et al., 2020), it should be noted that these methods require a large training sample size in the training phase—which can be viewed as a limitation for this type of brain study with a limited dataset. There is a large variety of deep-learning architectures that can be used in brain imaging data, including a convolutional neural network (CNN), a recurrent neural network (RNN), and an auto encoder (AE). Hosseini et al. (2020) used a CNN deep learning structure for the localization and prediction of epileptogenicity based on EEG and rs-fMRI data. In an investigation by Si et al. (2020), a CNN-wise transfer learning technique combined with high angular resolved diffusion imaging (HARDI) and NODDI data were used for the detection of juvenile myoclonic epilepsy. A CNN model based on rs-fMRI data was trained for the classification of pediatric refractory epilepsy (Nguyen et al., 2021).

A random forest classification model works based on an ensemble learning method and voting for multiple unpruned decision trees. The bootstrap sample of the original dataset generates a random distribution of the samples for each decision tree. By eliminating the overfitting problems in decision-making trees, a random forest model is able to improve the predicting accuracy. In the context of epilepsy, a random forest algorithm has been used in various studies (Paldino et al., 2017a, b; Vasta et al., 2018). For example, Park and Ohn (2019) used a random forest classifier for estimating the seizure frequency in TLE through structural MTI features. In addition to classification tasks, the random forest method has been used for the determination of feature importance and selection (Fallahi et al., 2020). Other classification algorithms have also been applied in epilepsy studies, including XGBoost (Torlay et al., 2017), a naïve Baysian classifier (Hwang et al., 2019b), Adaboost (Park et al., 2020), and a quadratic discriminant analysis (Chiang et al., 2015).

#### Regression Model

Support vector regression (SVR) is known as the most widely used regression model for the prediction of continuous variables. SVR is used to find an optimal hyperplane that deviates from the training data as little as possible, such as linear regression. Unlike linear regression (in which the algorithm is aimed at minimizing the observed training errors), an SVR model measures the error on the basis of data points rather than a “margin of tolerance.” SVR has shown a very good performance in regression analyses for neuroimaging (Hwang et al., 2020; Sone et al., 2021).

Gaussian process regression (GPR) is a non-parametric Bayesian method for regression tasks. GPR works based on a probability distribution of possible values. Both SVR and GPR models have been used for estimating the brain age in epilepsy (Pardoe et al., 2017; Hwang et al., 2020; Sone et al., 2021). Logistics regression is a statistical model that models the association between predictor variables and a categorical response variable. The output of a logistic regression model is a probability value that falls into a 0–1 range, but with the use of a classification cut-off (i.e., probability of 0.5), logistic regression can be used for classification tasks (Pustina et al., 2015; Peter et al., 2018). Logistic regression has been widely used a binary classifier in epilepsy studies (Ahmed et al., 2015; Mahmoudi et al., 2018; Guo et al., 2020).

**FIGURE 3:**
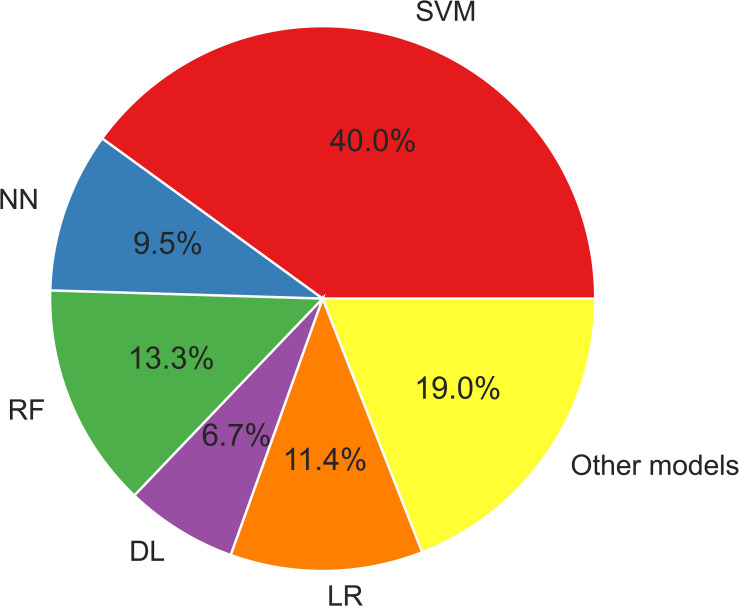
The ML models used in epilepsy studies. DL, deep learning; LR, logistic regression; NN, neural network; RF, random forest; SVM, support vector machine. Other models: XGBoost, LightGBM, CatBoost, decision tree, quadratic discriminant analysis.

Figure 3 displays the usage of the machine learning models described herein. SVM algorithms have been widely applied in epilepsy studies compared to other ML models. This is because SVM provides an optimal solution for solving a complex problem by using different kernels, which is appropriate for high-dimensional data and limited sample studies.

### Validation Strategies

Cross-validation is frequently used to evaluate the performance of ML models. The aim of cross-validation is to achieve an unbiased estimate of the classification/regressing performance and avoid overfitting by dividing the data into a training set and a test set. Data can be split once (i.e., split into a training set and a holdout test set) or several times (i.e., k-fold cross-validation). In the hold-out strategy, the data are randomly divided into a training set and an independent test set, and a training subset is used to train a prediction model (i.e., classification or regression) and optimize the respective parameters, whereas the independent test set is used to estimate the performance of the trained prediction model.

In the k-fold cross-validation strategy, data randomly split into k number of folds (i.e., k-subsets) and the learning process repeat k times such that for each iteration, k − 1 folds are used for training a prediction model, and the rest of the folds are used for a test. It should be noted that with the k-fold cross-validation strategy, there might be an overlap among the training subjects in different iterations (Noirhomme et al., 2014). Permutation tests can thus be used for assessing the statistical significance of k-fold cross-validation strategies (Noirhomme et al., 2014). In addition, fivefold and tenfold cross-validations have been suggested to provide a trade-off between bias and variance in ML models for brain imaging studies (Lemm et al., 2011). The leave-one-out strategy is a subtype of the k-fold cross-validation strategy in which the number of folds is equal to the number of samples; it is usually used for a small dataset. The k-fold cross-validation strategy has been widely used in epilepsy studies (Bharath et al., 2019; Beheshti et al., 2020a; Zhou et al., 2020; Sone et al., 2021).

## The Differentiation of Individuals With Epilepsy From Healthy Controls

A common application of machine learning for brain imaging in epilepsy is the differentiation between brains with epilepsy and healthy brains. As summarized in Table 1, various ML classifiers have achieved over 70–80% accuracy to successfully discriminate between individuals with epilepsy and healthy controls, using T1 images (Vasta et al., 2018; Chen et al., 2020; Park et al., 2020), diffusion MRI (Cantor-Rivera et al., 2015; Del Gaizo et al., 2017; Park and Ohn, 2019; Huang et al., 2020; Si et al., 2020), and functional MRI (Pedersen et al., 2015; Torlay et al., 2017; Wang et al., 2018a; Bharath et al., 2019; Hwang et al., 2019a, b; Zhou et al., 2020; Nguyen et al., 2021). Studies targeting TLE achieved ∼90% accuracy (Cantor-Rivera et al., 2015; Bharath et al., 2019; Chen et al., 2020; Huang et al., 2020), but it has been more challenging to identify idiopathic generalized epilepsy (IGE), and only ∼75% accuracy has been obtained for this task (Wang et al., 2018a; Si et al., 2020). Though these impressive investigations yielded evidence of the potential of machine learning in epilepsy, the clinical usefulness of the findings might be limited, since a differentiation between individuals with epilepsy and healthy subjects is not a major role of neuroimaging.

**TABLE 1:** ML applications used for the differentiation of individuals with epilepsy and healthy subjects.

References	Subjects	Imaging modality	Imaging features	Classifiers	Main outcomes
Pedersen et al. (2015)	9 LGS, 14 HC	rs-fMRI	EC, ReHO	MVPA	ACC = 0.957 for LGS vs. HC
Cantor-Rivera et al. (2015)	17 TLE (8 R, 9 L), 19 HC	T1, T2, DTI	T1/T2 signals, FA, MD	SVM	ACC = 0.889 for TLE vs. HC
Del Gaizo et al. (2017)	32 left TLE, 36 HC	DKI	FA, MD, MK	SVM	ACC = 0.82 for TLE vs. HC by MK
Torlay et al. (2017)	16 FE, 39 HC	Task-fMRI	BOLD	XGBoost	AUC = 0.91 for FE vs. HC
Wang et al. (2018a)	14 IGE-GTCS (P), 30 HC	T1, rs-fMRI	Morph (GMV), fALFF	SVM	ACC = 0.74–0.83 for IGE vs. HC
Vasta et al. (2018)	23 PNES, 21 HC	T1	Morph (SBM, GMV)	RF	ACC = 0.745 on average for PNES vs. HC
Hwang et al. (2019a)	55 TLE (14 R, 26 L, 2 B, 13 U)	T1, rs-fMRI	Morph (SBM, GMV), FC	SVM	ACC 0.734 for TLE vs. HC. Association between cognitive slowing and MRI
Hwang et al. (2019b)	69 TLE, 59 HC	rs-fMRI	FC, ALFF, fALFF	SVM, LDA, naïve Baysian classifier	ACC ∼0.83, AUC ∼0.90 for TLE vs. HC
Bharath et al. (2019)	42 TLE-HS (18 R, 19 L, 5 B)	rs-fMRI	IC	SVM	ACC = 0.975 for TLE vs. HC. Correlation of network with clinical variables
Park and Ohn (2019)	42 TLE (18 R, 24 L), 45 HC	T1, DTI	Morph (GMV, WMV), FA	RF	ACC = ∼80% for TLE vs. HC, ∼70% to predict seizure frequency
Huang et al. (2020)	59 TLE (P), 70 HC	DKI	FA, MD, MK	SVM	ACC = 0.908 for TLE vs. HC. CNN was used for feature extraction.
Park et al. (2020)	66 TLE (35 R, 31 L), 65 HC	T1	Morph (radiomics)	SVM, LR, AdaBoost	AUC = 0.84 for LTLE vs. HC or RTLE vs. HC
Si et al. (2020)	15 JME, 15 HC	HARDI, NODDI	Network measures	CNN	ACC = 0.752, AUC = 0.839 for JME vs. HC
Zhou et al. (2020)	74 TLE-HS (37 R, 37 L), 74 HC	T1, rs-fMRI	Morph (GMV, WMV, SBM), ALFF, ReHO	SVM	ACC = 84.1 for LTLE vs. HC, 72.9 for RTLE vs. HC (when all features combined)
Chen et al. (2020)	22 TLE-HS (6 R, 16 L), 15 HC	T1	Morph (VBM)	SVM	AUC = 0.870 for LHS vs. HC, 0.976 for RHS vs. HC, 0.902 for HS vs. HC
Nguyen et al. (2021)	63 DRE (P), 259 HC	rs-fMRI	Temporal latency	CNN	ACC = 0.74, AUC = 0.86 for DRE vs. HC

*ACC, accuracy; AUC, area under the ROC curve; B, bilateral; BOLD, blood-oxygen-level-dependent; CNN, convolutional neural network, DKI, diffusion kurtosis imaging, DRE, drug-resistant epilepsy, DTI, diffusion tensor imaging, EC, eigenvector centrality, FA, fractional anisotropy, fALFF, fractional amplitude of low-frequency fluctuations, FC, functional connectivity, FE, focal epilepsy, fMRI, functional MRI, GMV, gray matter volumes, HARDI, high-angular-resolution diffusion imaging, HC, healthy controls, HS, hippocampal sclerosis, IC, independent components, IGE-GTCS, idiopathic generalized epilepsy with generalized tonic-clonic seizures, L, left, LDA, linear discriminant analysis, LGS, Lennox-Gastaut Syndrome, LR, logistic regression, MD, mean diffusivity, MK, mean kurtosis, Morph, morphological features, MVPA, multivariate pattern analysis, NODDI, neurite orientation dispersion and density imaging, (P), pediatric cases, PNES, psychogenic non-epileptic seizures, R, right, ReHO, regional homogeneity, RF, random forest, rs, resting-state, SBM, surface-based morphometry, SVM, support vector machine, TLE, temporal lobe epilepsy, U, unknown, VBM, voxel-based morphometry; WMV, white matter volume.*

## Lateralization of TLE Foci

TLE is the most prevalent form of adult epilepsy and often causes drug-resistant seizures (Engel, 1996). There is clear evidence that surgical resection is more effective for refractory TLE than a continued use of anti-epilepsy drugs (Wiebe et al., 2001), and the accurate lateralization of the focus side in TLE is thus clinically important and one of the main targets of neuroimaging research in epilepsy.

As listed in Table 2, there have been various ML approaches to the lateralization of TLE foci, using T1-weighted images, diffusion MRI, FLAIR images, 18F-FDG-PET, or a combination of these (Focke et al., 2012; Keihaninejad et al., 2012; Kerr et al., 2013a, b; An et al., 2014; Hosseini et al., 2014; Chiang et al., 2015; Pustina et al., 2015; Yang et al., 2015; Kamiya et al., 2016; Fang et al., 2017; Mahmoudi et al., 2018; Peter et al., 2018; Bennett et al., 2019; Beheshti et al., 2020a, c; Fallahi et al., 2020; Hosseini et al., 2020). The applications of these approaches to cases without visually detectable lesions, i.e., so-called MRI-negative TLE, would be particularly beneficial in clinical settings by providing further clues to the focus beyond the conventional approaches. Although the current accuracy of ML lateralization for MRI-negative TLE seems not as high as that for MRI-positive cases (> 98%) (Bennett et al., 2019), this approach has achieved > 75% accuracy, which would be acceptable in clinical practice (Keihaninejad et al., 2012; Bennett et al., 2019; Beheshti et al., 2020a, c).

**TABLE 2:** ML applications used for the lateralization of TLE foci.

References	Subjects	Imaging modality	Imaging features	Classifiers	Main outcomes
Keihaninejad et al. (2012)	80 TLE (60 HS, 20 NL), 28 HC	T1	Morph (GMV)	SVM	ACC = 0.96 for HS vs. HC, 0.91 for NL vs. HC, 0.94 for lateralization of TLE-NL
Focke et al. (2012)	38 TLE-HS (18 R, 20 L), 22HC	T1, DTI, T2	Morph (GMV, WMV), T2 signal, FA, MD	SVM	ACC = 0.88–0.93 for LTLE vs. RTLE vs. HC
Kerr et al. (2013b)	73 TLE (34 R, 39 L), 32 NES	FDG-PET	PET signal	MLP	ACC = 0.82–0.88 for TLE vs. NES, 0.76 for lateralization of TLE
Kerr et al. (2013a)	73 TLE (34 R, 39 L), 32 NES, 30 HC	FDG-PET	PET signal	MLP	ACC = 0.81 for lateralization of TLE. No effect of the choice of control group.
Hosseini et al. (2014)	76 TLE	T1, FLAIR	Morph (GMV), FLAIR signal	SVM, MLPNN	ACC = 0.82 for lateralization of TLE.
An et al. (2014)	32 TLE (15 R, 17 L), 34 HC	DTI	FA	SVM	ACC = 0.918–0.941 for TLE vs. HC, 0.906 for lateralization of TLE
Yang et al. (2015)	12 TLE (5 R, 7 L)	rs-fMRI	FC, Network measures	SVM	ACC = 0.83 for lateralization of TLE
Pustina et al. (2015)	58 TLE (30 R, 28 L)	T1, DTI, FDG-PET	Morph (SBM, GMV), FA, PET signal	LR	ACC > 0.95 for lateralization of TLE by PET
Chiang et al. (2015)	24 TLE (10 R, 14 L)	rs-fMRI	Network measures	QDA	AUC = 0.96 for lateralization of TLE
Kamiya et al. (2016)	44 TLE (15 R, 29 L), 14 HC	DTI	Network measures	SVM	ACC ∼0.80 for TLE vs. HC, LTLE vs. RTLE
Fang et al. (2017)	43 TLE (21 R, 22 L), 39 HC	DTI	SC	SVM	ACC > 0.90 for TLE vs. HC, < 70% for LTLE vs. RTLE
Mahmoudi et al. (2018)	68 TLE (54 HS, 14 NL)	T1	Morph (GMV)	LR, SVM	ACC > 0.90 for lateralization in both TLE-HS and TLE-NL
Peter et al. (2018)	17 TLE (11 R, 6 L), 23 HC	FDG-PET	PET signal	LR	AUC = 0.80 for lateralization of TLE
Bennett et al. (2019)	104 TLE (82 MRI+, 22 NL)	T1, T2, FLAIR	Morph (GMV), T2, FLAIR signals	SVM	AUC = 0.981 for MRI+, 0.842 for NL, for lateralization of TLE. RFC was used for feature extractions.
Beheshti et al. (2020a)	42 TLE-NL (19 R, 23 L), 34 HC	FLAIR	FLAIR signal	SVM	ACC = 0.75 for 3 groups, 0.762 for lateralization of TLE
Fallahi et al. (2020)	35 TLE (14 R, 21 L)	rs-fMRI	Network measures	RF, SVM	AUC up to 0.91 for LTLE vs. RTLE
Beheshti et al. (2020b)	56 TLE-NL (27 R, 29 L)	FDG-PET	PET signal	SVM	ACC = 0.964 for lateralization of TLE
Hosseini et al. (2020)	9 TLE (5 R, 4 L)	rs-fMRI	FC	CNN, SVM	Successful lateralization when combined with fMRI and EEG

*ACC, accuracy; AUC, area under the ROC curve; CNN, convolutional neural network; DTI, diffusion tensor imaging; FA, fractional anisotropy; FC, functional connectivity; GMV, gray matter volumes; HC, healthy controls; HS, hippocampal sclerosis; L, left; LR, logistic regression; MD, mean diffusivity; MLP, multilayer perceptron; MLPNN, multilayer perceptron neural network; Morph, morphological features; MRI+, MRI-positive; NES, non-epileptic seizure; NL, no lesion; QDA, quadratic discriminant analysis; R, right; RF, random forest; rs-fMRI, resting-state functional MRI; SBM, surface-based morphometry; SC, structural connectivity; SVM, support vector machine; TLE, temporal lobe epilepsy; WMV, white matter volume.*

## Identification of Epileptogenic Foci, Particularly in Focal Cortical Dysplasia (FCD)

The accurate localization of the epileptogenic focus is highly relevant for successful epilepsy surgery (Rathore and Radhakrishnan, 2015), which may remediate drug-resistant focal epilepsy. Structural MRI in particular plays a major role in the visual detection of focus lesions, and it has been widely used in clinical practice for epilepsy (Bernasconi et al., 2019). As seen in Table 3, there have been various applications of machine learning for lesion identification to improve the detection rate or to develop automated algorithms (Hong et al., 2014; Ahmed et al., 2015; Rudie et al., 2015; El Azami et al., 2016; Adler et al., 2017; Jin et al., 2018; Tan et al., 2018; Wang et al., 2018b; Mo et al., 2019; Alaverdyan et al., 2020; Lee et al., 2020a; Wagstyl et al., 2020; Snyder et al., 2021; Zhang et al., 2021), which would be concordant with the seizure onset zone detected by intracranial EEG (Kanber et al., 2021). Focal cortical dysplasia (FCD), which is a common cause of intractable epilepsy, is characterized by abnormal cortical thickness, blurring of the gray-white matter junction, and T2/FLAIR hyperintensity (Bernasconi et al., 2019). It is thus reasonable that an accurate ML diagnosis was usually achieved by structural MRI (such as T1 or FLAIR imaging, often using surface-based methods) rather than functional MRI (Table 3). More recent studies tend to use combined data from multimodal imaging, whereas earlier studies used only T1WI (Table 3). Differential diagnoses such as FCD type I vs. II and FCD vs. tumor were also reported (Hong et al., 2016; Guo et al., 2020).

**TABLE 3:** ML applications used for the detection of epileptogenic foci, including FCD.

References	Subjects	Imaging modality	Imaging features	Classifiers	Main outcomes
Hong et al. (2014)	33 FCD, 44 HC, 11 TLE	T1	Morph (SBM), signal intensity	LDA	Sens. 71% Spec. 95% to automatically detect FCD
Rudie et al. (2015)	169 EPI (85 HS, 84 NL)	T1	Morph (SBM, VBM)	SVM	ACC = 0.81 for HS vs. NL
Ahmed et al. (2015)	31 FCD, 62 HC	T1	Morph (SBM)	LR, IRLS	Detection in 6 of 7 MRI positive cases, 14 of 24 MRI-negative
El Azami et al. (2016)	11 FE, 77 HC	T1	Texture parameters	SVM	AUC > 0.90 to detect epileptogenic lesions
Hong et al. (2016)	41 FCD-FLE, 41 HC	T1	Morph (SBM)	SVM	ACC = 98% for Type I vs. II, approximately 90% for lateralization, 82–92% to predict seizure outcome
Adler et al. (2017)	22 FCD, 28 HC	T1, FLAIR	Morph (SBM), FLAIR signal	NN	AUC around 0.7–0.8 using various feature combinations
Wang et al. (2018b)	12 FCD	DTI, T2	FA, MD, VR, T2 signal	GPML, SVM	AUC = 0.76 to automatically detect FCD by GPML
Jin et al. (2018)	61 FCDII, 155 HC, 15 HS	T1	Morph (SBM)	NN	AUC = 0.75 to detect FCD
Tan et al. (2018)	28 FCD, 23 TLE	T1, FDG-PET	Morph (SBM), GM intensity, PET signal	SVM	Sens. = 0.93 to detect FCD, when combined MRI and PET
Mo et al. (2019)	80 TLE-HS (39R, 41L), 80 HC	T1	Visual features, Morph	SVM, E-net LR	AUC around 0.98–0.99 for TLE-HS vs. HC, 96% detection rate for visually negative HS
Lee et al. (2020a)	46 FCD, 35 HC	T1, FLAIR, rs-fMRI	Morph (SBM), FLAIR signal, Gradient, Ratio, fALFF	Consensus clustering (unsupervised)	Four relevant structural profiles (WM, GM, GM and WM, GM-WM interface) were identified
Wagstyl et al. (2020)	34 FCD (P), 20HC	T1, FLAIR	Morph (SBM), FLAIR signal	NN	Sens. = 0.74, Spec = 1.00 to detect FCD, concordance with SOZ based on SEEG
Alaverdyan et al. (2020)	21 FE, 75HC	T1, FLAIR	Signals	SVM, RSN	Sens. = 0.62 to detect anomaly lesion
Guo et al. (2020)	56 FCD, 40 GNTs	T1, T2, FLAIR	Visual assessment	RF, SVM, DT, LR, XGBoost, LightGBM, and CatBoost	AUC = 0.934 for FCD vs. GNTs by RF-based ML when combined MRI and clinical info
Snyder et al. (2021)	15 FCD, 30 HC	T1, T2, FLAIR	Morph (SBM), signal intensity	Normative model	80% Sens., 70% Spec. to detect FCD
Zhang et al. (2021)	201 TLE (P), 24 Ctrl (lymphoma)	FDG-PET	Radiomics	CNN	AUC = 0.93, ACC = 0.90 to detect epileptogenic focus

*ACC, accuracy; AUC, area under the ROC curve; CNN, convolutional neural network; DT, decision tree; DTI, diffusion tensor imaging; E-net LR, elastic net logistic regression; EPI, epilepsy; FA, fractional anisotropy; fALFF, fractional amplitude of low-frequency fluctuations; FCD, focal cortical dysplasia; FE, focal epilepsy; FE, focal epilepsy; FLE, frontal lobe epilepsy; GBM, gradient boosting algorithm; GM, gray matter; GNTs, glioneuronal tumors; GPML, Gaussian processes for machine learning; HC, healthy controls; HS, hippocampal sclerosis; IRLS, iterative-reweighted least squares; L, left; LDA, linear discriminant analysis; LR, logistic regression; MD, mean diffusivity; Morph, morphological features; NL, no lesion; NN, neural network; (P), pediatric cases; R, right; RF, random forest; rs-fMRI, resting-state functional MRI; RSN, regularized Siamese neural network; SBM, surface-based morphometry; SEEG, stereotactic EEG; SOZ, seizure onset zone; SVM, support vector machine; TLE, temporal lobe epilepsy; VBM, voxel-based morphometry; VR, volume ratio; WM, white matter.*

## Prediction of Clinical Outcomes

There are also various ML applications for more direct associations with clinical outcomes than lesion/focus detection (Table 4). A major trend in this section is the prediction of postsurgical seizure freedom (Bernhardt et al., 2015; Memarian et al., 2015; Munsell et al., 2015; He et al., 2017; Gleichgerrcht et al., 2018; Taylor et al., 2018; Gleichgerrcht et al., 2020; Larivière et al., 2020; Kini et al., 2021; Sinha et al., 2021), in light of the clinical importance. Most of the studies reported 70–90% accuracy for the prediction of seizure outcomes after resection surgery. Other studies presented approximately 85% accuracy for the identification of responders to vagus nerve stimulation (VNS) (Ibrahim et al., 2017; Mithani et al., 2019). In terms of surgery, ML methods were also applied and generated good predictive values for postsurgical functional deficit (Lee et al., 2020b), lateralization of the language hemisphere (Gazit et al., 2016), and optimal planning for laser surgery (Li et al., 2019).

**TABLE 4:** Machine learning applications used to predict clinical outcomes in epilepsy.

References	Subjects	Imaging modality	Imaging features	Classifiers	Main outcomes
Paldino et al. (2014)	32 MCD (P)	DTI	FA, MD	RF	Sens. = 1.00, Spec. = 0.954 to predict language impairment
Amarreh et al. (2014)	20 EPI (P), 29 HC	DTI	FA, MD, AD, RD	SVM	ACC > 90% for EPI vs. HC, > 75% to discriminate remission
Memarian et al. (2015)	20 TLE (10 R, 10 L)	sMRI	Morph (SBM)	SVM	ACC = 95% to predict seizure outcome after surgery, when combined with other clinical info/EEG
Munsell et al. (2015)	70 TLE, 43 HC	DTI	Network measures	SVM	ACC = 0.80 for TLE vs. HC, 0.70 to predict seizure outcome after surgery
Bernhardt et al. (2015)	141 TLE	T1	SBM	k-means clustering (unsupervised)	Four data-driven distinct classes of TLE, associated with histopathology and seizure outcome
Gazit et al. (2016)	76 EPI	Task-fMRI	BOLD	LR	89% concordance with WADA to lateralize language hemisphere
Ibrahim et al. (2017)	21 EPI (P)	rs-fMRI	FC	SVM	ACC = 0.86–0.88 to predict VNS response
He et al. (2017)	56 TLE (30 R, 26 L), 28 HC	rs-fMRI	Network measures	SVM	ACC = 0.70 to predict seizure outcome after surgery
Paldino et al. (2017b)	45 FE (P)	rs-fMRI	Network measures	RF	*R* = 0.95 to predict epilepsy duration
Paldino et al. (2017a)	30 FE (P)	rs-fMRI	Network measures	RF	ML revealed no effect of motion parameters or general amnesia during the scan for IQ prediction
Zhang et al. (2018)	24 FE	rs-fMRI	FC, Network measures	RF	0.49 of fractional variation to predict IQ
Gleichgerrcht et al. (2018)	50 TLE	DTI	Network measures	NN	PPV = 0.88, NPV = 0.79 to predict seizure outcome after surgery
Taylor et al. (2018)	53 TLE (30 L, 23 R)	DTI	Network measures	SVM, E-net LR	ACC = 0.792 to retrospectively predict seizure outcome after surgery
He et al. (2018)	50 TLE (25 R, 25 L), 30 HC	fMRI (task, rs)	Network measures	RF	∼100% prediction for verbal fluency, improvement from traditional methods
Mithani et al. (2019)	56 EPI (P)	DTI	FA	SVM	ACC = 0.83–0.89 to predict VNS responders, when combined with MEG
Yao et al. (2019)	287 EPI	Routine MRI	Visual assessment	DT, RF, SVM, LR, XGBoost	AUC = 0.979 to predict AED responders, 0.918 for early responders when combined with clinical info/EEG.
Paldino et al. (2019)	27 FE (P)	rs-fMRI	Network measures	RF	0.34 fractional variation to predict IQ
Zhang et al. (2019)	117 AVM	T2	Location, Radiomics	LASSO	ACC around 0.800 to predict epilepsy occurrence
Li et al. (2019)	10 TLE-HS (5 R, 5 L)	T1	Morph (SBM, etc.)	RF, LR	Accurately predict the optimal trajectories for LITT
Ernst et al. (2019)	46 GAD, 34 VGKC, 48 HC	T1	Morph (GMV, etc.)	DT	Spec. = 0.87, Sens. = 0.80 for GAD vs. VGKC
La Rocca et al. (2020)	53 TBI	T1	Network measures	RF	AUC = 0.75 to predict seizure occurrence after TBI
Gleichgerrcht et al. (2020)	168 TLE	T1, DTI	Network measures	NN	AUC = 0.88 to predict seizure outcome after surgery by BC
Höller et al. (2020)	9 TLE, 19 MCI, 4 SCI, 18 HC	T1	Morph (GMV, etc.)	SVM	Sens/Spec = 0.70–0.90 to predict cognitive decline over time, when combined with MRI, EEG, Neuropsychology.
Larivière et al. (2020)	30 TLE-HS (R 19, L 11), 57 HC	T1, DTI, rs-fMRI	Connectivity distance	LR	ACC = 0.76 to predict seizure outcome after surgery
Mazrooyisebdani et al. (2020)	27 TLE (R9, L18), 85 HC	rs-fMRI	FC	SVM, SVR	ACC = 0.81 for TLE vs. HC. R = 0.61–0.75 to predict neuropsychology
Akeret et al. (2020)	923 brain tumors	T1, FLAIR	Anatomical features	DT, GLM, RF, GBM, NN, SVM, GAM	AUC = 0.79, ACC = 0.72 to predict seizure occurrence when combined with clinical info
Lee et al. (2020b)	89 FE (P)	DTI	WM tract	CNN	ACC = 0.92 to predict functional deficit after surgery
Wang et al. (2020)	205 LGG-related EPI	T2WI	Signal, shape, etc.	Novel radiomic nomogram	AUC = 0.863 to predict epilepsy type
Sinha et al. (2021)	51 TLE, 29 HC	T1, DTI	Network measures	SVM	AUC = 0.84 to predict seizure outcome after surgery
Kini et al. (2021)	89 TLE	FDG-PET	PET signal	RF	ACC = 0.71 to predict seizure outcome after surgery

*ACC, accuracy; AD, axial diffusivity; AUC, area under the ROC curve; AVM, arteriovenous malformation; BC, betweeness centrality; BOLD, blood-oxygen-level-dependent; CNN, convolutional neural network; DT, decision tree; DTI, diffusion tensor imaging; E-net LR,; EPI, epilepsy; FA, fractional anisotropy; FC, functional connectivity; FC, functional connectivity; FE, focal epilepsy; fMRI, functional MRI; GAD, anti-GAD-related encephalitis; GAM, generalized additive model; GBM, gradient boosting algorithm; GLM, generalized linear model; GMV, gray matter volumes; HC, healthy controls; L, left; LASSO, least absolute shrinkage and selection operator; LGG, low-grade gliomas; LITT, laser interstitial thermal therapy; LR, logistic regression; MCD, malformation of cortical development; MCI, mild cognitive impairment; MD, mean diffusivity; Morph, morphological features; NN, neural network; (P), pediatric cases; R, right; RD, radial diffusivity; RF, random forest; rs, resting-state; SBM, surface-based morphometry; SCI, subjective cognitive impairment; sMRI, structural MRI; SVM, support vector machine; SVR, support vector regression; TBI, traumatic brain injury; TLE, temporal lobe epilepsy; VGKC, anti-VGKC-related encephalitis.*

Cognitive dysfunctions in epilepsy were also shown to be predicted by ML methods (Paldino et al., 2014, 2017a, 2017b, 2019; He et al., 2018; Zhang et al., 2018; Höller et al., 2020; Mazrooyisebdani et al., 2020). Functional neuroimaging and/or network measurement are often used for this prediction (Paldino et al., 2017a, b, 2019; He et al., 2018; Zhang et al., 2018; Mazrooyisebdani et al., 2020). Other ML applications included predicting drug responsiveness (Amarreh et al., 2014; Yao et al., 2019), acquiring epileptogenicity (Zhang et al., 2019; Akeret et al., 2020; La Rocca et al., 2020; Wang et al., 2020), and the differentiation of types of autoantibodies (Ernst et al., 2019).

## Regression Models (Brain-Age Prediction)

Another trend in the field of neuroimaging and machine learning is regression models, which are often used for the prediction of brain aging (Cole and Franke, 2017). Human brains change with aging, and this may also be associated with various neuropsychiatric diseases. To investigate the relationships between brain aging and epilepsy, several research groups have the regression model technique (Pardoe et al., 2017; Chen et al., 2019; Hwang et al., 2020; Sone et al., 2021).

In general, an increase in the age of the brain by ∼4–10 years has been reported (Table 5), which is consistent with recent evidence of disease progression or tau deposition in epilepsy (Tai et al., 2016; Galovic et al., 2019). The increased brain age in individuals with epilepsy seems to be associated with longer disease duration, early onset age, and/or psychiatric comorbidity (Pardoe et al., 2017; Chen et al., 2019; Sone et al., 2021).

**TABLE 5:** ML applications for brain-age prediction in epilepsy.

References	Subjects	Imaging modality	Imaging features	Classifiers	Main outcomes
Pardoe et al. (2017)	136 FE (94 DR, 42 ND), 74 HC, (2001 HC for model)	T1	VBM	GPR	+4.5 years in DR-FE, but non-significance in ND-FE.
Chen et al. (2019)	35 TLE (17 R, 18 L), 37 HC (300 HC for model)	DSI	GFA, AD, RD, MD, NG, NGO, NGP	GPR	+10.9 years in RTLE, +2.2 years in LTLE Correlation with onset age, duration, seizure frequency
Hwang et al. (2020)	104 TLE, 151 HC	T1, rs-fMRI	SBM, FC	SVR	+6.6 years in structural MRI, +8.3 years in functional MRI
Sone et al. (2021)	318 EPI, 1192 HC	T1	VBM	SVR	>+4 years in almost all forms of epilepsies +10.9 years in TLE with psychosis

*AD, axial diffusivity; DR, drug-resistant; DSI, diffusion spectrum imaging; EPI, epilepsy; FC, functional connectivity; FE, focal epilepsy; GFA, generalized fractional anisotropy; GPR, Gaussian process regression; HC, healthy controls; L, left; MD, mean diffusivity; ND, newly diagnosed; NG, non-Gaussianity; NGO, NG orthogonal; NGP, NG parallel; R, right; RD, radial diffusivity; rs-fMRI, resting-state functional MRI; SBM, surface-based morphometry; SVR, support vector regression; TLE, temporal lobe epilepsy; VBM, voxel-based morphometry.*

## Methodological Aspects and Future Directions

As described, the current ML applications for epilepsy imaging are diverse in terms of the targeted epilepsy syndromes, imaging modalities, feature extractions, and ML strategies. Multimodal imaging is a recent trend in epilepsy research, and it may provide comprehensive information (Sidhu et al., 2018). Accordingly, there has been an increase in the number of ML studies using multiple imaging modalities, especially in recent years. However, as shown in Tables 1–5, each study group seems to have some tendencies regarding the choice of imaging modalities, which may have led to the diversity of research in this field.

Feature extraction is another significant factor in the diversity of this research. While most studies have used a region-of-interest (ROI) to extract imaging features, the choice of atlases for ROIs varies. For example, some investigations used traditional automated anatomical labeling (AAL) (Fallahi et al., 2020; Si et al., 2020; Kini et al., 2021), and a different atlas was used in other studies (Gleichgerrcht et al., 2018, 2020). Zhang et al. (2019, 2021) used radiomics as a novel method to extract imaging data, and this might provide greater usefulness than conventional methods (Gillies et al., 2016). For better clinical applications, we should develop and validate consistent methodologies, since these factors may directly affect the prediction of outcomes and the algorithm itself.

Regarding the ML algorithms, more recent studies have tended to use deep-learning methods such as a CNN (Hosseini et al., 2020; Lee et al., 2020b; Si et al., 2020; Nguyen et al., 2021; Zhang et al., 2021). Another important point about methodology is the shortage of studies using unsupervised classification; indeed, only two studies adopted unsupervised clustering (Bernhardt et al., 2015; Lee et al., 2020a). Given the potentials of unsupervised clustering for finding hidden patterns in unlabeled data, further studies using this method are needed to uncover data-driven information.

## Conclusion

Machine learning is an emerging trend in the field of neuroimaging in epilepsy, and promising results have been obtained in many studies. The diversity in terms of targeted epilepsy syndromes, imaging modalities, feature extractions, and ML algorithms provides an extra challenge. Recent trends include the use of deep learning, multimodal imaging, and regression models, and additional investigations using unsupervised clustering are desired. For better clinical applications, consistent methodologies must be developed and validated.

## Author Contributions

Both authors listed have made a substantial, direct and intellectual contribution to the work, and approved it for publication.

## Conflict of Interest

The authors declare that the research was conducted in the absence of any commercial or financial relationships that could be construed as a potential conflict of interest.
